# Bioactive effects of nonthermal argon-oxygen plasma on inorganic bovine bone surface

**DOI:** 10.1038/s41598-020-75195-2

**Published:** 2020-10-21

**Authors:** Chengzan Wu, Kai Ma, Hongmei Zhao, Qian Zhang, Yanshan Liu, Na Bai

**Affiliations:** 1 The Affiliated Hospital of Qingdao University, Qingdao, 266003 China; 2 School of Stomatology, Qingdao University, Qingdao, 266003 China; 3 Department of Prosthodontics, The Affiliated Hospital of Qingdao University, No.16 Jiangsu Rd, Qingdao, 266003 China; 4 Department of Oral and Maxillofacial Surgery, The Affiliated Hospital of Qingdao University, Qingdao, 266003 China

**Keywords:** Biochemistry, Chemical biology, Chemistry, Materials science

## Abstract

As a commonly used bone substitute material in the clinic, inorganic bovine bone has the characteristics of osteoconduction but not osteoinduction. This study aimed to treat inorganic bovine bone using nonthermal argon-oxygen plasma (NTAOP) to obtain greater bioreactivity for enhancing adhesion, proliferation and differentiation of mouse preosteoblast MC3T3-E1 cells. In this study, inorganic bovine bone was activated by NTAOP, and the surface characteristics were analyzed. MC3T3-E1 cells were then seeded onto the surface of inorganic bovine bone. Cell morphology, proliferation and osteogenic differentiation were examined. There was no obvious change in the surface morphology of specimens between the two groups. Regarding the elemental composition of the material, the amount of surface carbon was reduced, whereas oxygen, phosphorus and calcium levels were increased in the NTAOP group. Further studies showed that the NTAOP groups performed better than their untreated counterparts in terms of supporting cell proliferation and differentiation. Inorganic bovine bone treated with NTAOP can promote preosteoblast adhesion, proliferation and differentiation.

## Introduction

Bone grafting is often used surgery used to repair bone defects caused by trauma, tumors, and congenital malformations. Autografting is considered the best way to achieve the optimal expected effect; however, there are many insurmountable disadvantages in clinical application of bone grafting due to the problems of demand, due to increasing surgical trauma, and surgical complications. In addition, allografts possess the risk of disease transmission and immunoreaction. As a result, the clinical applications of materials used as artificial bone substitutes have been rapidly developed^1,2^.

Inorganic bovine bone, a porous particle mainly composed of hydroxyapatite (HAP), is a frequently used bone substitute in the clinic. It can promote the growth of vessels and maintain the stability of blood clots, thus providing early nutrition for bone regeneration^3^. Nonetheless, as a bone substitute, it has osteoconductive ability but not osteoinductive ability^4^. As a competent bone graft material, good biocompatibility is a necessary condition. Although artificial bone substitutes are widely used in clinical practice, in human research, they display incomplete remodeling, and deposits can be frequently detected. Moreover, after implanting artificial bone substitutes in defective areas, most patients need 6 months or even longer to heal, which prolongs the time of later repair. Some studies show that the success of bone transplantation is also restricted by other factors, including host bone remodeling capacity, maximum material absorption, material toxicity and surgical infection^5^. Therefore, proper surface treatment of inorganic bovine bone material is one of the effective ways to improve its osteogenic efficiency and repair bone defects.

Nonthermal plasma is an electrically neutral, highly ionized gas composed of ions, electrons, excited molecules and atoms as well as neutral particles and radicals^6^, and it emits ultraviolet radiation^7^. Nonthermal plasma, which is stable at a low temperature, can directly treat living tissue through sterilization, coagulation, wound healing and tissue regeneration^6,8^. Recent studies^9^ have identified the potential advantages of nonthermal plasma treatment for bone and cartilage regeneration. However, the biological effects of nonthermal plasma treatment on inorganic bovine bone substitutes have not been studied.

Nonthermal plasma can modify the surface of inorganic bovine bone in a short time so that the surface wettability of specimens can be remarkably improved. Additionally, it also possesses the advantages of high efficiency, safety (free from secondary pollution), and simplicity, and there is no need for expensive vacuum equipment and a tedious time-consuming operation because nonthermal plasma can be used under normal pressure. The above advantages of nonthermal plasma make it capable of treating the surface of bone substitute materials under normal temperature and pressure conditions so that it can possibly improve the bioactivity properties of bone substitute materials before implantation.

The objective of this study was to promote the bioactivity of inorganic bovine bone surfaces via NTAOP treatment. After NTAOP treatment, we chemically characterized inorganic bovine bone. Moreover, mouse preosteoblasts MC3T3-E1 were cultured and seeded onto specimens to examine the biological activities of inorganic bovine bone and determine whether nonthermal argon-oxygen plasma can improve the biological characteristics of inorganic bovine bone and enhance its osteogenic capacity.

## Materials and methods

### Activation of inorganic bovine bone

**Figure 1:**
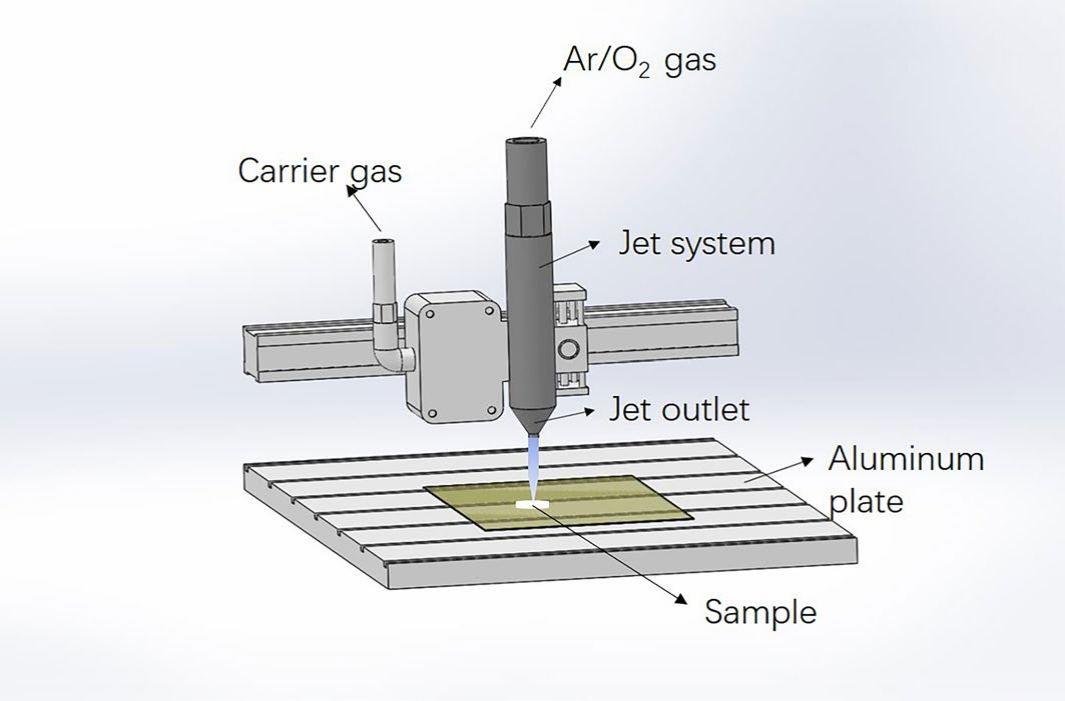
Schematic illustration of the Atmospheric Pressure Plasma System. In this system, 95% Ar and 5% O2 were used as inputting gases to generate plasma.

An Atmospheric Pressure Plasma System Model AS400 + PFW10 (PLASMA Treat GmbH, Steinhagen, Germany) was used for plasma activation. A schematic diagram of the system is shown in Fig. 1. It is a glow-discharge plasma workspace that is 33 cm by 12.5 cm, and it was powered by radio-frequency power (19 kHz) and a matched network. The device parameters were adjusted to a voltage of 320 V and current of 0.3 A, and the gas flowed to the jet orifice at a rate of 500 L/h. The substrate was fixed on an aluminum plate, and the plasma jet was controlled by a three-dimensional motion system. In addition, the inorganic bovine bone block (HEAL-ALL, 5 mm × 10 mm × 20 mm, ZH-BIO) was fixed at a position that was 2.5 cm away from the nonthermal plasma jet nozzle and was treated for 60 s with NTAOP produced from argon (Ar, 95%) and oxygen (O_2_, 5%) as the output gas^10^. The processed samples were chemically characterized and were evaluated in vitro for their biocompatibility.

### Surface morphology observation

A scanning electron microscope (SEM) was used to observe and compare the bone block surface morphology before and after NTAOP treatment. Specifically, an inorganic bovine bone that was subjected to surface treatment according to the predetermined parameters was classified as the NTAOP group, while the untreated group received no treatment. Prior to observation, the bone block surface was coated with palladium to obtain better imaging quality, and the samples were observed under different magnifications. The accelerating voltage was 10 kV, and the magnifications were 2000× and 10000×.

### X-ray photoelectron spectroscopy (XPS)

An X-ray photoelectron spectrometer (Thermo Escalab 250XI, Thermo Fisher Scientific, USA) was employed for the tests. A monochromatic Al Kα source was used as an X-ray source (hv = 1253.6 eV). The working voltage was set to 12.5 kV, the filament current was 16 mA, and 10 circulating signals were collected. Moreover, the pass energy for photoelectrons was set to 40 eV with steps of 0.1 eV. The binding energy scales for the specimens were calibrated by setting the value of the carbon bonded to either carbon or hydrogen to 284.60 eV. The elemental and chemical analyses of the two groups were carried out using an X-ray photoelectron spectrometer, and high-resolution [C1s], [O1s], [Ca2p] and [P2p] spectra were collected.

### Early cell adhesion observed by SEM and CLSM

Bone block was prepared in a 24-well plate. Then, MC3T3-E1 cells were seeded into the material at a density of 2 × 10^6^ cells/well and then were placed into the incubator to culture for 3 h; subsequently, the cell culture medium was removed. Later, cells were washed with phosphate-buffered saline (PBS) twice, fixed with 2.5% glutaraldehyde, dehydrated with an ethanol concentration gradient (30%, 50%, 70%, 90% and 100%) for 15 min each, and cell morphology was observed under SEM after metal spraying.

Adhesion behavior and cytoskeletal arrangement of MC3T3-E1 cells on the surface of bone blocks were investigated using confocal laser scanning microscopy (CLSM) (Leica TCS SP5 II, Leica SPE, Germany). To observe actin, 24 h after seeding the cells we performed fluorescent staining for actin using TRITC phalloidin (actin filament, red color) (CA1610, Beijing Solarbio Science & Technology Co., Ltd). 4′, 6-diamidino-2-phenylindole (DAPI) (cell nucleus, blue color) (C0065, Beijing Solarbio Science & Technology Co., Ltd) was used as a counterstaining. Image was collected in triplicate and presented with representative pictures. Quantification of MC3T3-E1 cells coverage areas was using image analysis software (Image J, NIH, Bethesda, ML).

### In vitro cell proliferation

Bone block was prepared in a 96-well plate. Cell density was adjusted to 3 × 10^5^ cells/mL. Then, 100 μl of cell suspension was seeded onto the material surface; then, two hours later, 100 μl of culture media was slowly added along the well wall edge. After 2 h, 4 h and 1 day, cell growth on the material surface was observed under a microscope. On days 1, 3, and 5, 200 μl of fresh culture media was added into each well to replace the old culture media. Subsequently, 10 μl of CCK-8 reagent was added to each well. The 96-well plate was placed in an incubator to incubate for two hours, and the absorbance was read at 450 nm with an automatic enzyme-linked immunosorbent assay reader (BIOBASE-EL 10A, SHANDONG).

### In vitro osteogenic differentiation

Bone block was prepared in a 96-well plate. Cell density was adjusted to 5 × 10^5^ cells/ml. Then, 100 μl of cell suspension was seeded onto the surface of the material and cultured with osteogenesis-supplementing factors. After culturing for 7 and 14 d, cells were washed with phosphate-buffered saline (PBS) three times, and 120 μl of lysate buffer was added into each well, followed by repeated thaw-freezing treatments to break the cell membrane. Then, the cell lysate was collected according to the alkaline phosphate (ALP) kit instructions. The optical density (OD) value was detected using an automatic enzyme-linked immunosorbent assay reader at 405 nm, and the ALP activity was converted according to a standard formula.

### Statistical analysis

All experiments in this study were repeated three times (n = 3). SPSS statistical software was used for analysis. The experimental data were expressed as the mean ± standard deviation and tested by one-way analysis of variance (ANOVA). Values of P < 0.05 were considered statistically significant.

## Results

### Surface morphology analysis

**Figure 2:**
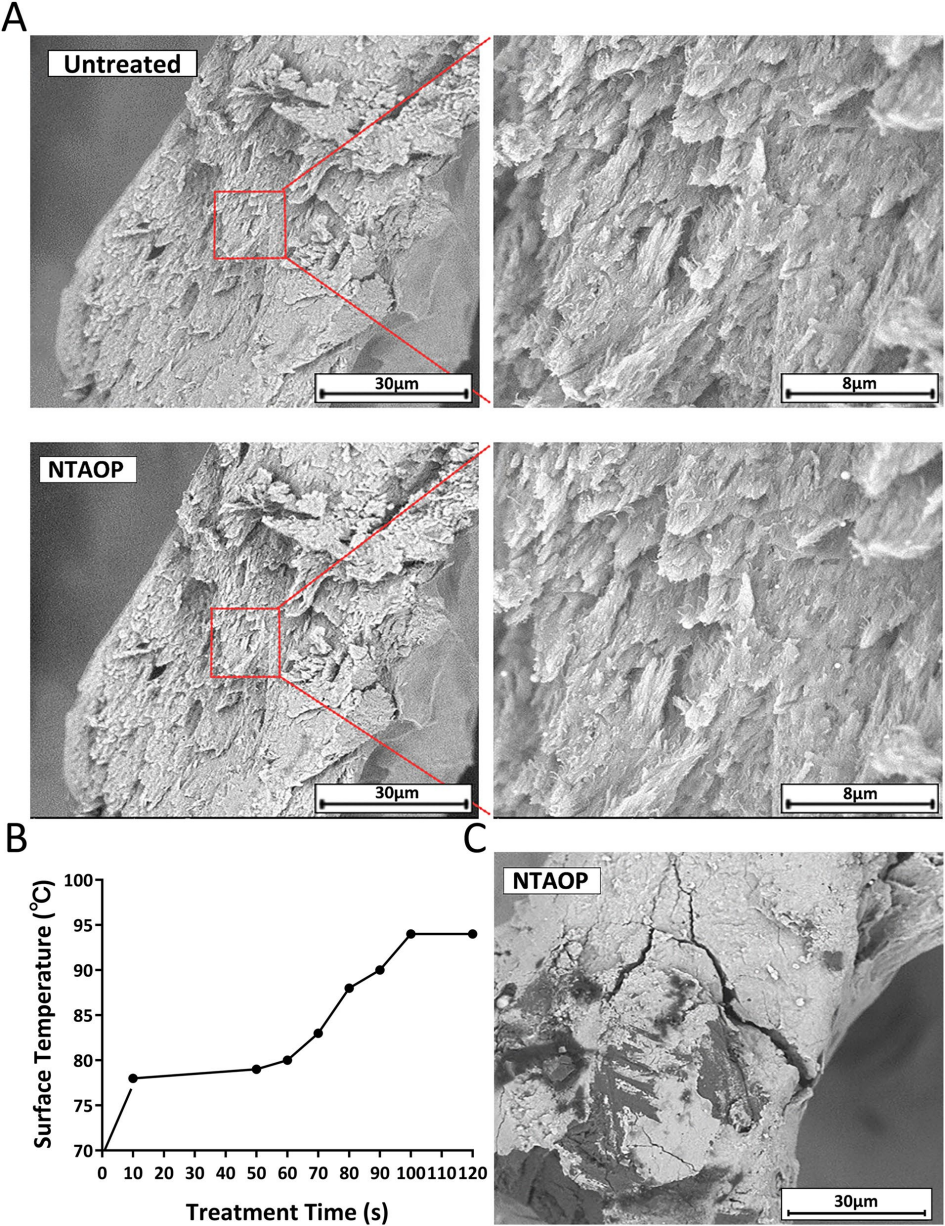
Surface morphology of samples. Scale bars represent 30 μm and 8 μm. (**A**) SEM images of samples show that there was no obvious change in surface morphology before and after NTAOP treatment. (**B**) Relationship between surface temperature and treatment time. (**C**) SEM images of samples show that when the treatment time was 90 s, the surface of the bone block was dry and cracked.

There was no obvious change in surface morphology between the untreated group and the NTAOP-treated group. The SEM results suggested that treating the surface with plasma did not cause noticeable injury on the bone block surface, which was still observed to be coarse, containing macroscopic and microscopic structures (Fig. 2A).

The preliminary results from a pre-experiment show that the treatment time is closely related to the surface temperature and morphology of the bone. When the treatment time was 60 s, the surface temperature of the bone block was approximately 80 °C, and the surface morphology of the bone block revealed no obvious change. When the treatment time was changed from 60 to 90 s, the surface temperature of the bone block slowly increased by approximately 15 °C, the surface of the bone block dried up and cracked, and the internal connection structure broke (Fig. 2B,C). Therefore, to ensure that NTAOP exerts its activation ability and does not cause a change in bone surface morphology, the treatment time selected for future experiments was 60 s.

### XPS results

**Figure 3:**
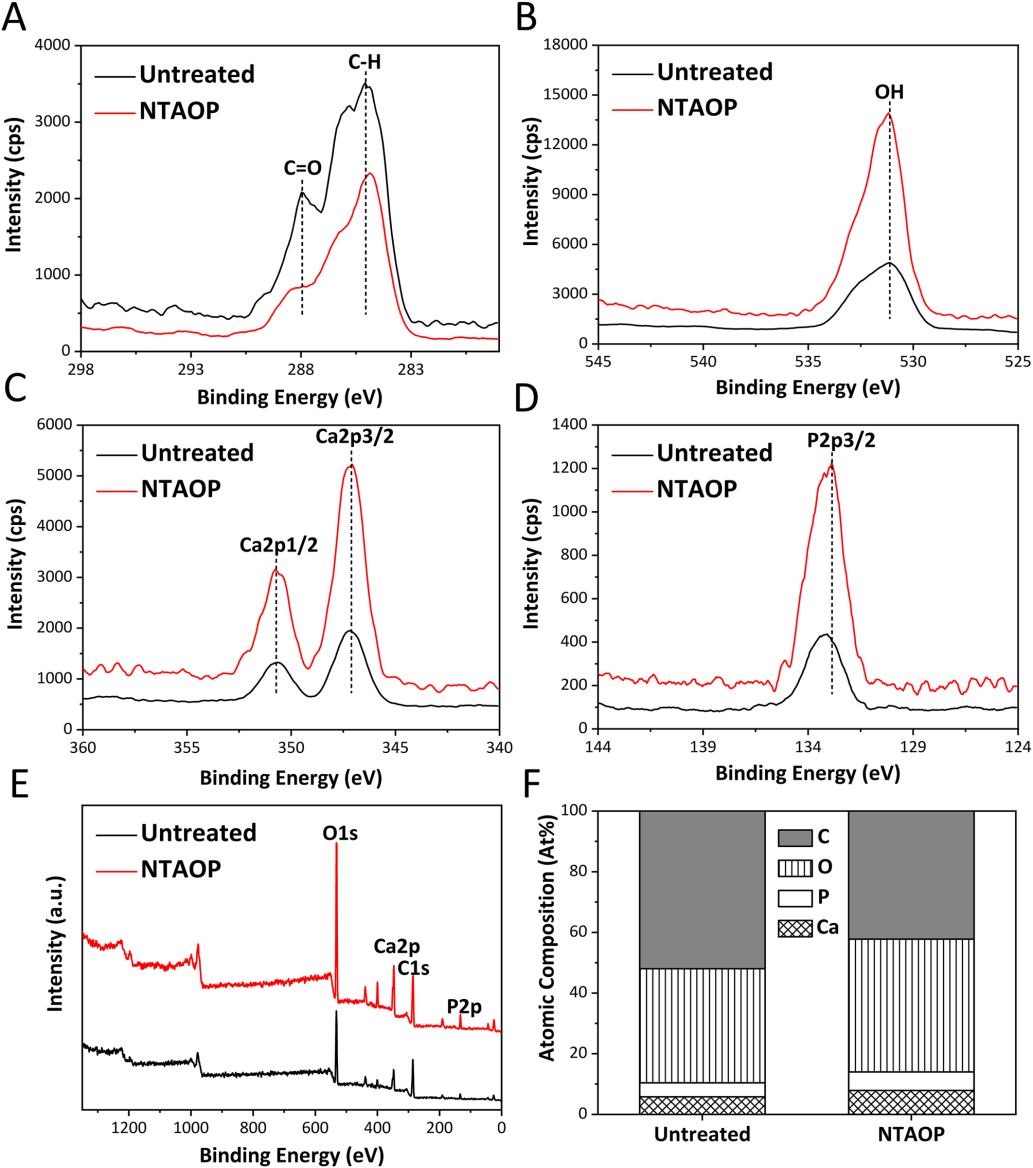
Chemical composition of the samples measured by XPS. (**A**) C1s spectra, (**B**) O1s spectra, (**C**) Ca2p spectra and (**D**) P2p spectra were determined. (**E**) High-resolution XPS spectra of samples before or after NTAOP treatment. (**F**) Atomic composition of each element of the samples before and after NTAOP treatment.

As shown in Fig. 3F, the element composition in both groups was largely the same, but there were some differences. After NTAOP treatment, the percentage of C on the bone surface was reduced, while those of elements O, P and Ca were increased.

Figure 3E shows the XPS spectrum of the material before and after NTAOP treatment. The XPS intensity results of each element suggested that after NTAOP treatment, the peak for the hydroxyl group (–OH) on specimens at a binding energy of 531.2 eV increased (Fig. 3B,E). The observed changes can increase the hydrophilicity of the specimens as well as the surface energy. In addition, (Fig. 3C–E) shows the peaks for the Ca2p 1/2 and Ca2p 3/2 (347 eV) and P2p 3/2 (132.8 eV) groups, and all peaks increased in intensity after NTAOP. After NTAOP treatment, the intensity of carbon–oxygen (C–O) at a binding energy of 288.6 eV and hydrocarbon (–CH) at a binding energy of 285 eV (C1) was apparently decreased, revealing that NTAOP effectively removed organic impurities from the bone surface (Fig. 3A,E).

### Cell adhesion observed by SEM and CLSM

**Figure 4:**
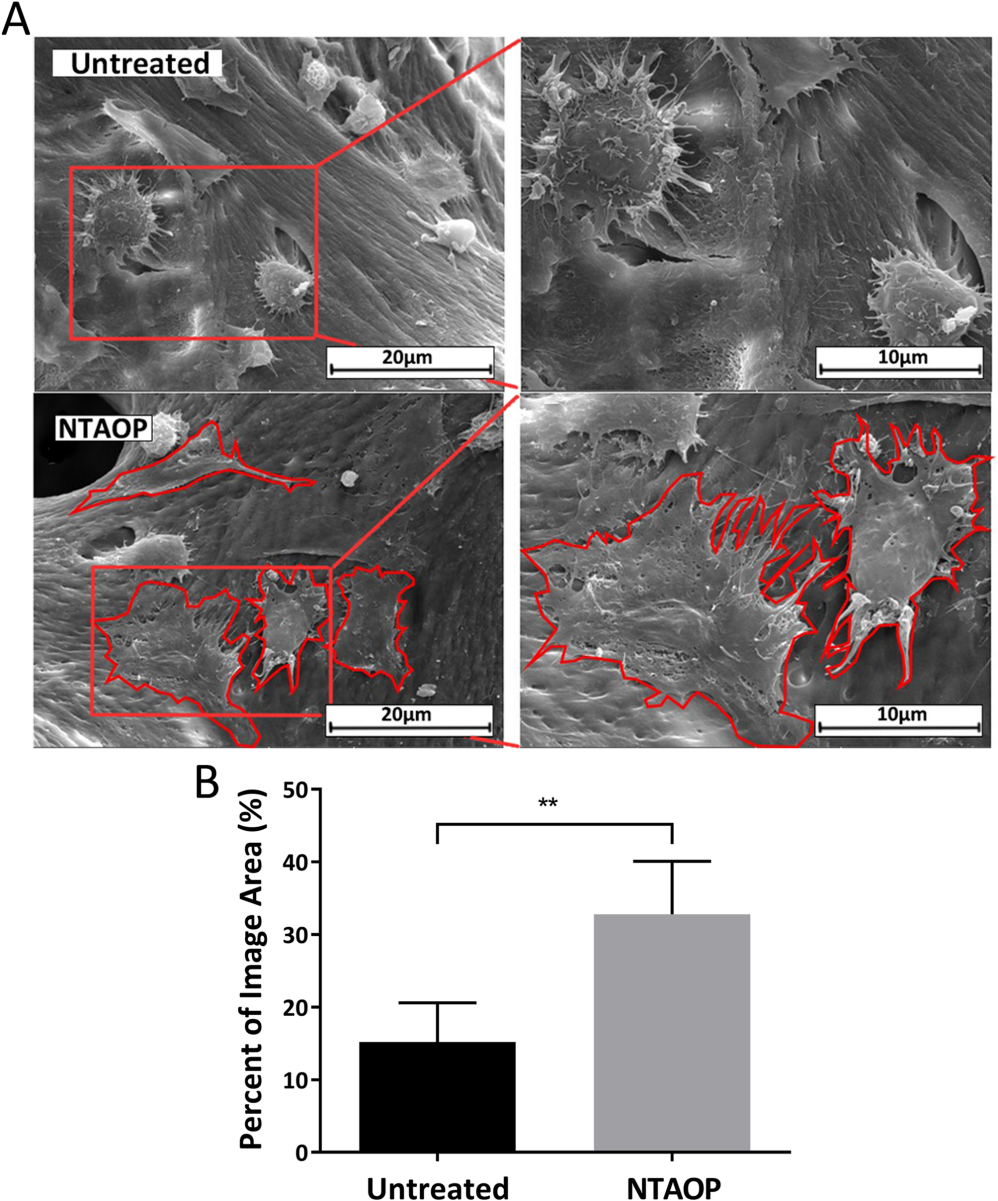
(**A**) SEM images of attached MC3T3-E1 cells in the untreated group and NTAOP group. Scale bars represent 20 μm and 10 μm. (**B**) Quantification of the MC3T3-E1 cell adhesion area in each group. The percentage of adherence area of MC3T3-E1 cells in the NTAOP group increased dramatically compared to that in the untreated group (**P < 0.05).

Under 20 μm magnification, most cells in the untreated group were found to be spherical and adhered to the material surface (Fig. 4A). Under 10 μm magnification, cells in the untreated group appeared to be spherical with the formation of pseudopods, but there was no obvious spreading (Fig. 4A). Under 20 μm magnification, most cells in the NTAOP group appeared to be fibroblastoid or polygonal in shape (Fig. 4A Red line marker). Under 10 μm magnification, cells in the NTAOP group appeared to have sufficiently spread, as long, thick intercellular contacts protruded around the cells (Fig. 4A red line). Figure 4B presents the percentage of osteoblasts that adhered in an area on the bone surface before and after NTAOP treatment, and the difference was statistically significant (P < 0.05).

**Figure 5:**
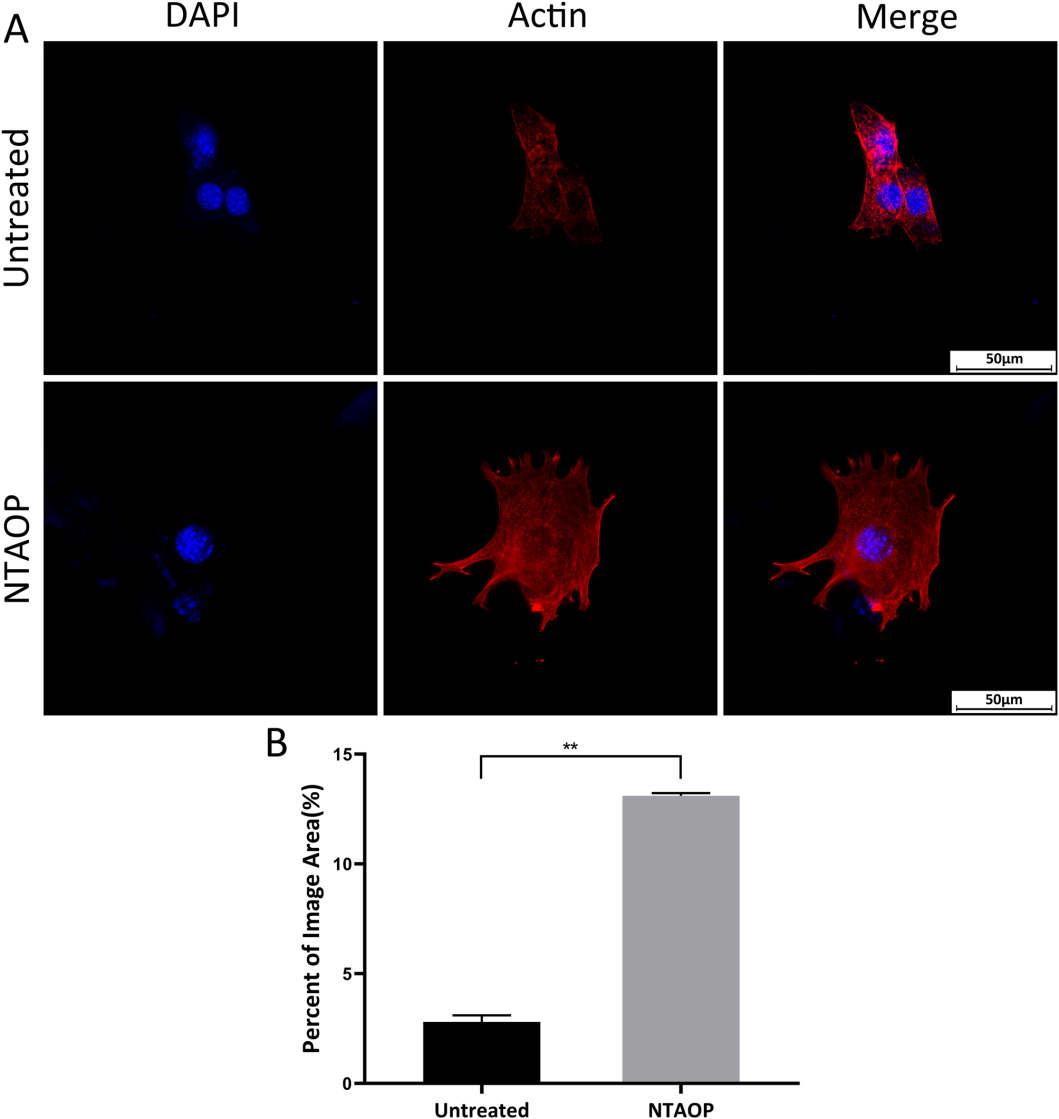
(**A**) CLSM images of actin cytoskeleton of MC3T3-E1 cells. Scale bar represents 50 μm. (**B**) Quantification of the MC3T3-E1 cell adhesion area in each group. The percentage of adherence area of MC3T3-E1 cells in the NTAOP group increased dramatically compared to that in the untreated group (**P < 0.05).

Figure 5 showed the staining of actin of MC3T3-E1 cells in all samples. After 24 h of incubation, the actin fluorescent staining pictures of the cells showed that the cells in the NTAOP group were fibroblastoid in shape and fully stretched compared to the untreated group. The cells in the untreated group were mostly round and insufficiently stretched, indicating insufficient cytoskeletal development. (Fig. 5A). The quantitative results showed that the cell coverage area was significantly increased after NTAOP treatment (Fig. 5B).

### Cell proliferation detection

**Figure 6:**
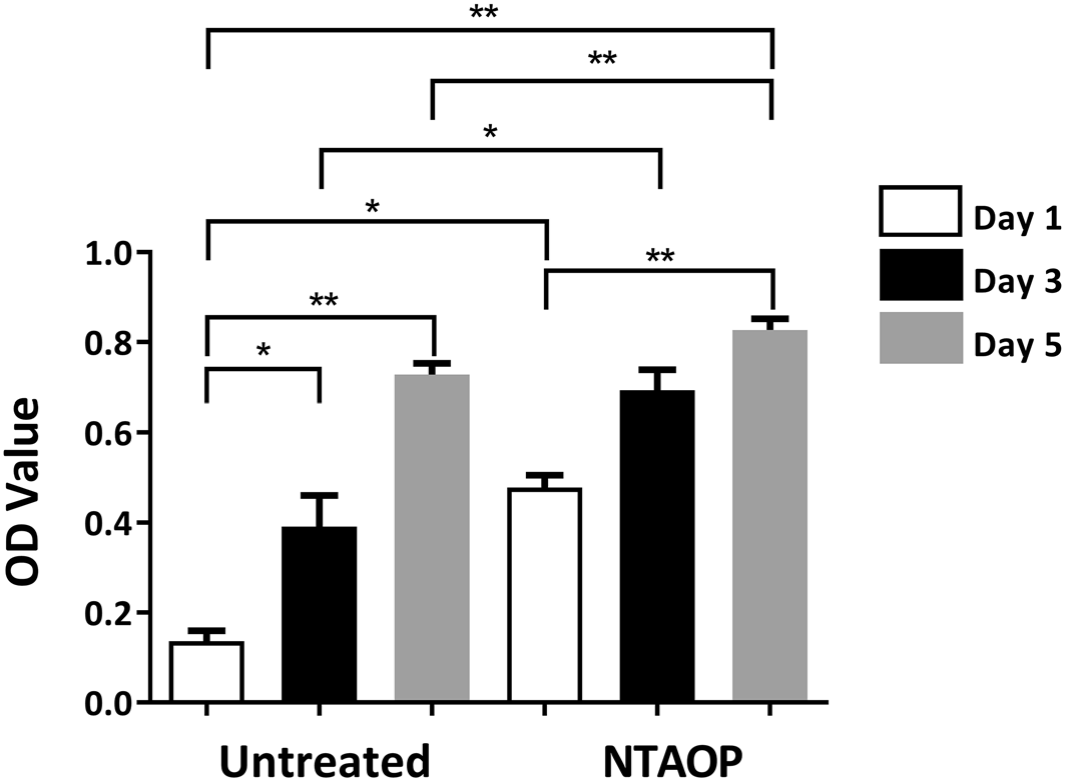
The proliferation of MC3T3-E1 cells in the untreated group and NTAOP group. CCK-8 assay results showed that after 1, 3, and 5 days of culture, the rates of proliferation increased substantially in the NTAOP group compared with the untreated group (*p < 0.05, and **, < 0.01).

The CCK-8 results suggested that over time, the proliferating cell numbers on the bones in both groups gradually increased. The differences in the proliferating cell number in the untreated group between 1 and 3 days, as well as between 1 and 5 days, were statistically significant. Meanwhile, the differences in the proliferating cell number between 1 and 5 d in the NTAOP group, as well as between 1 and 5 days in the untreated group, were statistically significant. Moreover, the proliferating cell numbers on the bone surface activated by NTAOP on days 1, 3 and 5 were greater than those on the untreated surface, and the differences were statistically significant (Fig. 6) (*P < 0.05, and **P < 0.01).

### Detection of cell differentiation

**Figure 7:**
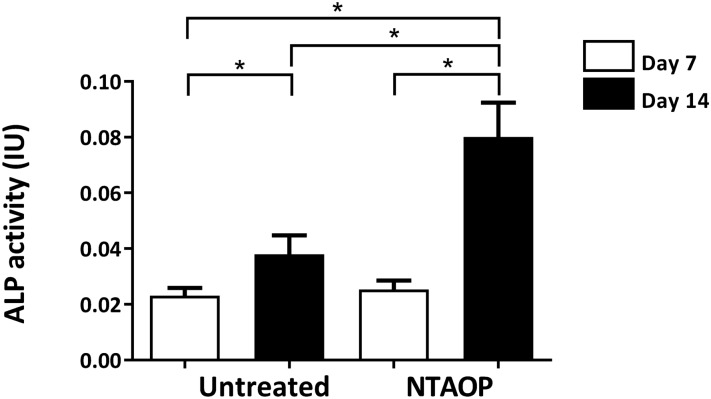
The differentiation of MC3T3-E1 cells in the untreated group and NTAOP group. ALP assay results showed that after 14 days of culture, the rates of ALP activity increased dramatically in the NTAOP group compared with the untreated group (*p < 0.05).

According to the ALP results, as time increased, the ALP activity in cells from the two groups of bone blocks gradually increased, and the differences from 7 to 14 days in both the untreated and NTAOP groups were statistically significant (*P < 0.05). On days 7 and 14, the cell ALP activity on the bone surface activated by NTAOP was higher than that on the untreated surface, and the difference between the two groups on day 14 was statistically significant (Fig. 7) (*P < 0.05).

## Discussion

Inorganic bovine bone is a type of bone substitute that has been commonly used in recent years. It has good biocompatibility and is similar to mineralized human bone in morphology and chemical structure. It currently extensively applied in the clinic and has attained favorable osteogenic effects. However, it has been discovered in clinical practice that this material is associated with the drawback of not being easily completely adsorbed by the body^11,12^. Consequently, improving the osteogenic effect of inorganic bovine bone has become a research focus in recent years. Schmitt C et al.^13^ modified inorganic bovine bone powder with BMP-2 and VEGF protein to give it osteoinductive function and enhance its osteogenic effect. However, the protein synthesis process is complex and expensive, and it may cause an immune response or infection after implantation, which limits its clinical application. Tátrai et al.^14^ utilized a cyclic RGD-containing peptide polymer to modify inorganic bovine bone to enhance its osteogenic effect, and their results suggested that the physical binding of inorganic bovine bone with the peptide polymer promoted cell adhesion and differentiation, but it did not promote cell proliferation; further, the experiment is complicated and difficult to execute, which has limited its clinical adoption. As a result, searching for a simple and efficient surface treatment to promote the osteogenic effect of inorganic bovine bone has practical significance.

Plasma is a physical form mainly constituted by free electrons and charged ions, which are distributed extensively throughout the universe. It is frequently regarded as the fourth state of matter, being referred to as the plasma state^15^. Plasma has been extensively applied in material surface processing, including cleaning and activation^16,18^. In addition, nonthermal plasma has the advantages of being active at a low temperature and being highly efficient, which have caused it to attract extensive attention in recent years in biomedical fields^17^. Duske et al.^18^ treated different surfaces of titanium discs with nonthermal argon plasma and observed that the spreading of osteoblasts was noticeably improved. Some existing studies indicate that when noble gases, such as argon (Ar) and oxygen, are mixed at a certain ratio and used as metastable noble gas species, they can carry a large amount of energy, which can result in the formation of reactive oxygen species (ROS) through energy transfer reactions in the nonthermal plasma environment to substantially increase the material surface energy^19,20^. Therefore, we hypothesized that treatment of an inorganic bovine bone surface with nonthermal plasma produced by the mixed gases of argon and oxygen could produce a more ideal osteogenic reaction than what has been previously produced.

Inorganic bovine bone we used is a three-dimensional porous structure obtained by deproteinized treatment prepared from cancellous bones of calves. In this study, when the treatment time was 60 s, the surface temperature of the bone block was approximately 80 °C, and the surface morphology of the bone block revealed no obvious change. When the treatment time was more than 60 s, the surface temperature of the bone block will continue to rise, the surface of the bone block dried up and cracked, and the internal connection structure broke. Therefore, it is necessary to maintain the time of NTAOP treatment within a certain range to obtain a lower temperature. At this temperature, the surface morphological observation results before and after NTAOP treatment suggest that there was no noticeable difference in the surface morphology of the untreated group and NTAOP group, indicating that NTAOP would not damage the inorganic bovine bone surface structure, which is consistent with previous results^21–23^.

The XPS results demonstrated that the peak intensities of oxygen, calcium, and phosphorus in the NTAOP group were increased compared with those in the untreated group, while the peak value of C was reduced relative to that in the untreated group. This might be because NTAOP treatment induced chemical changes on the surface of the material, which manifested as the reduction in hydrocarbon content as well as the increases in functional groups hydroxyl (–OH) and carboxyl (–COOH)^24,25^. Carbon, as a result of the unavoidable attachment of carbon-containing atmospheric components on the material surface and the adsorption of carbon on the surface, causes a decrease in biological activity^26^, thus affecting the combination of inorganic bovine bone and osteoblasts. In addition, the surface cleaning effect of plasma^27,28^ can effectively remove the carbon chemical residues on the surface of inorganic bovine bone, which is conducive to promoting the adhesion of osteoblasts. Franca et al. pointed out that it is important to remove these carbon chemical impurities from bioceramic materials because they will affect the mechanical properties of materials during bone regeneration^29^. Therefore, we believe that NTAOP can remove impurities from the surface of the material and promote its biological activity, which is confirmed by the following experimental results. The increase of oxygen content on the surface of the material is conducive to the improvement of its surface energy and ability to promote early cell adhesion, while the increase of calcium and phosphorus, which are important elements in bone tissue metabolism, is also conducive to the attachment and spread of cells^30^. However, after nonthermal plasma treatment, the surface chemical composition changes with time. Some studies have shown that the contact angle of a titanium disc surface after nonthermal plasma treatment increases with exposure to air over time. This may be due to the short half-life of functional groups on the surface of the material and the reaction between the surface oxide film and hydrocarbons in the air, resulting in a decrease in the hydrophilicity of the material^31^.

The SEM results of early osteoblast adhesion suggested that the osteoblasts on the surface of the untreated group were spherical, as no obvious spreading was observed. Compared with the untreated group, the osteoblasts on the bovine bone surface activated by NTAOP were polygonal and flat, and they demonstrated excellent adhesion and stretching properties. Moreover, from the material surface, great numbers of cell processes and mature cytoskeletal development were also observed. This result indicated that the inorganic bovine bone activated by NTAOP contributed to early osteoblast adhesion. This might be due to the increase in oxygen and the decrease in carbon on the material surface after plasma treatment. Some studies show that the elevation in oxygen content and the decrease in carbon content can increase the hydrophilicity of the material surface and the surface energy, while the material surface plays a vital role in promoting early cell adhesion^32,33^.

In this study, the CCK-8 cell proliferation assay results suggest that there was noticeably more cell proliferation on bovine bone surfaces treated with NTAOP than there was in the untreated group. This indicates that the activated material surface efficiently enhanced the osteoblast proliferation capacity, which might be related to the increase in material surface activity. A large number of studies indicate that the surface characteristics of biological material directly affect cell reaction, thus impacting the growth rate and quality of new tissue^34^. In the early stage of osteoblast differentiation, ALP is expressed, which is why it is regarded as an early marker of extracellular matrix maturation. Therefore, ALP detection is a simple and economical method for specifically detecting osteoblasts. The ALP detection results suggest that the osteogenic activity in the NTAOP group on days 7 and 14 was higher than that in the untreated group, indicating that the material surface activated by plasma had a certain effect in promoting bone formation.

Inorganic bovine bone is a type of artificial bone substitute that has been extensively applied in fields of implantation and GBR (guided bone regeneration) due to its similarity in chemical properties to human bone. In this experiment, inorganic bovine bone was activated by NTAOP, and the results showed that the treated inorganic bovine bone partially promoted cell adhesion, proliferation and differentiation without changing the material surface morphology or its chemical composition. We believe that the inorganic bovine bone activated by NTAOP shows favorable prospects for enhancing osteogenic capacity, but this should be further proven in animal experiments and clinical studies.

## Conclusion

In this study, a simple, fast and effective method was proposed for the activation of inorganic bovine bone. NTAOP-activated inorganic bovine bone was stripped of carbon and oxygen-enriched. Inorganic bovine bone treated with NTAOP can promote preosteoblast adhesion, proliferation and differentiation. This indicates that NTAOP treatment is a promising technique with prospects for supporting inorganic bovine bone activation by improving its osteogenic capacity.
